# Diet contributes to microbiome and niche differentiation among three sympatric cervids

**DOI:** 10.3389/fmicb.2026.1980313

**Published:** 2026-10-14

**Authors:** Yuxiang Liang, Wentao Zhang, Feifei Yang, Yilin Zhang, Ruitao Wei, Chenbing Chu, Jiale Sun, Heng Bao, Weiping Li

**Affiliations:** 1 Heilongjiang Wildlife Institute, Harbin, China; 2 College of Wildlife and Protected Area, Northeast Forestry University, Harbin, China

**Keywords:** diet, microbiome, moose, niche differentiation, red deer, roe deer

## Abstract

**Background:**

Niche differentiation is a fundamental mechanism facilitating the coexistence of sympatric species, yet the extent to which dietary partitioning is associated with gut microbial differentiation remains poorly understood in wild cervids.

**Methods:**

Here, we investigated winter dietary composition and fecal microbiome communities of three sympatric cervid species—moose (*Alces alces*), red deer (*Cervus elaphus*), and roe deer (*Capreolus pygargus*)—in Youhao National Nature Reserve, northeastern China. A total of 55 fresh fecal samples were collected in January 2024, including 19 moose, 7 red deer, and 29 roe deer samples. Dietary composition was characterized using rbcL DNA metabarcoding, while microbiome communities were assessed using full-length 16S rRNA gene sequencing.

**Results:**

Our results showed the three cervid species exhibited distinct dietary patterns, moose showed a relatively narrow woody-plant-dominated diet, whereas red deer and roe deer exhibited higher dietary diversity, and roe deer exhibited broader use of shared plant resources; microbiome communities also differed significantly among host species, with moose showing the clearest separation from red deer and roe deer in community composition and predicted microbial metabolic potentials; Procrustes analysis revealed significant concordance between dietary composition and microbiome community, and redundancy analysis also indicated that dietary plant composition explained a significant proportion of variation in microbiome community.

**Conclusion:**

These findings demonstrate that trophic differentiation among sympatric cervids is accompanied by coordinated variation in gut microbial community structure and predicted functional potential. The results support a close ecological association between dietary niche differentiation and host-associated microbiome variation, while causal and directional mechanisms require confirmation through longitudinal, experimental, and direct functional studies.

## 1 Introduction

Niche differentiation is the core ecological theory to explain the long-term stable coexistence of sympatric species (Schoener, 1974). According to the competitive exclusion principle, species with highly similar niches inevitably undergo niche differentiation under limited resource conditions (Hardin, 1960). In recent years, researchers have conducted multi-dimensional investigations into the mechanisms by which sympatric species coexist (Weber and Strauss, 2016; Atwood et al., 2019; Si et al., 2025; Kihwele et al., 2025). Differentiated resource use across spatial, temporal and trophic mitigates interspecific competition and maintains stable community structure and function (Schoener, 1974; Chesson, 2000). One key indicator for understanding the coexistence of species in the same area is the differentiation of nutritional ecological niches (diet). Dietary differentiation reduces competition for food resources, which is important for the coexistence of multiple species (Hutchinson, 1959; Schoener, 1974; Kartzinel et al., 2019; Pansu et al., 2022). Traditional studies of nutritional ecological niches mostly rely on direct, on-site observation of animals foraging (Kleynhans et al., 2010; Bagchi and Ritchie, 2012), or microscopic histological methods to identify plant parts in faeces (Soininen et al., 2009; Iongh et al., 2011). However, these methods have limitations, including low taxonomic resolution, severe observer bias and the systematic underestimation of rare or highly digestible plant groups (Carrière, 2002). This obscures the subtle differences in dietary resources among sympatric herbivores and makes it difficult to accurately detect and reveal variations in the utilisation of food resources by different hosts (Kartzinel et al., 2015).

In recent years, gut microbiota have received a great deal of attention as the microscopic mediators of host trophic niche differentiation (Kartzinel et al., 2019; Moeller and Sanders, 2020). Regarded as the host’s second genome (Grice and Segre, 2012), gut microbial communities are critical determinants of host health, nutrition, development and fitness (Muegge et al., 2011; Holmes et al., 2012; Zhao et al., 2025). They not only participate in the degradation of plant cellulose, hemicellulose and secondary metabolites (Flint et al., 2012; Dearing and Kohl, 2017; McKenney et al., 2018), but also shape host adaptability to specific habitats by regulating energy metabolism and immune responses (Zhao et al., 2018; Zheng et al., 2020). Host physiological status, diet, lifestyle and immune environment collectively modulate the composition and functionality of gut microbiota (Zeng et al., 2025). Host phylogeny is another important source of variation in mammalian gut microbiota. A recent multispecies analysis showed that feeding habit and host phylogeny jointly structure gut microbial communities, with phylogenetic distance positively associated with microbiome dissimilarity, particularly among herbivores (Cheng et al., 2025). Therefore, interspecific microbial differences among sympatric herbivores may reflect not only dietary differentiation but also evolutionary relatedness among host species.

Studies have revealed that the composition and functionality of gut microbiota undergo dynamic shifts in response to fluctuations in host diet and habitat conditions (Kartzinel et al., 2019). Accordingly, gut microbial communities are a vital mechanism that enables hosts to cope with resource competition and achieve niche differentiation (Muegge et al., 2011; McKenzie et al., 2017). Therefore, divergence in the structure and function of gut microbiota reflects not only trophic niche partitioning among sympatric species, but also acts as a core functional mediator that drives and sustains such niche separation (Haonan et al., 2025; Zhou et al., 2025). Understanding the role of gut microbiota in forming and differentiating host trophic niches has profound theoretical significance for revealing the mechanisms that underlie the stable coexistence of multiple sympatric species at a microscale.

Moose (*Alces alces*) is the largest extant cervid species. In China, moose are found only in the Altai Mountains of Xinjiang, as well as in some areas of the Greater and Lesser Khingan Mountains, which represent the southernmost edge of their global distribution range (Sun et al., 2023). Red deer (*Cervus elaphus*) and roe deer (*Capreolus pygargus*) are long-term sympatric species coexisting with moose. The three species show substantial habitat overlap, creating the potential for interspecific competition for food resources.

Most relevant studies have focused solely on comparing the diets and gut microbiomes of one or two herbivore species (Yan et al., 2022; Anderson et al., 2025; Zheng et al., 2025). Constrained by traditional dietary identification techniques, such studies are unable to quantify subtle interspecific foraging divergence. There have been few systematic investigations into the link between the fine-scale foraging patterns of the three sympatric cervids and the compositional and functional characteristics of their gut microbial communities, in order to understand the mechanisms driving trophic niche differentiation.

Accordingly, this study aims to characterize similarities and differences in the dietary niches of the three sympatric cervid species and to examine their associations with the taxonomic structure and predicted functional profiles of the gut microbiome. To achieve this goal, we integrated plant dietary metabarcoding and 16S rRNA gene sequencing to characterize dietary compositions and the structural and functional traits of gut microbiomes across the three cervids. Specifically, this study addresses two questions: (1) What is the pattern of dietary differentiation among sympatric moose, red deer and roe deer? (2) Is dietary divergence associated with variation in the structure and predicted functional profiles of gut microbial communities among the three cervid species? The results of this study lay an important foundation for deciphering the niche differentiation of the three sympatric cervid species.

## 2 Methods

### 2.1 Sample collection

In January 2024, 55 fresh fecal samples were collected in Youhao National Nature Reserve, Yichun City, Heilongjiang Province, China, including 19 moose samples, 7 red deer samples and 29 roe deer samples (Figure 1). Fecal samples were collected during line-transect surveys covering the study area. Rather than concentrating sampling at a single fixed location, fresh fecal samples from the target cervid species were collected whenever suitable deposits were encountered along the survey transects. Consequently, the number of samples obtained for each species depended on the availability and encounter frequency of suitable fecal deposits during field surveys, resulting in unequal sample sizes among the three cervid species. Freshness was assessed based on fecal appearance, moisture, texture, and the absence of obvious environmental weathering. Intact fecal pellets were preferentially collected from the upper portion of each fecal pile, and material from the interior of the pellets was used whenever possible to minimize soil and environmental contamination. A new pair of disposable polyethylene (PE) gloves was used for each sample to prevent cross-contamination. Sampling was conducted from early morning to early afternoon, and freshly deposited feces were preferentially collected to minimize environmental degradation prior to preservation. To minimize the likelihood of repeated sampling from the same individual, only one fresh fecal sample was collected from each spatially discrete fecal sampling location. All specimens were stored at −80 °C until subsequent sequencing analyses.

**Figure 1:**
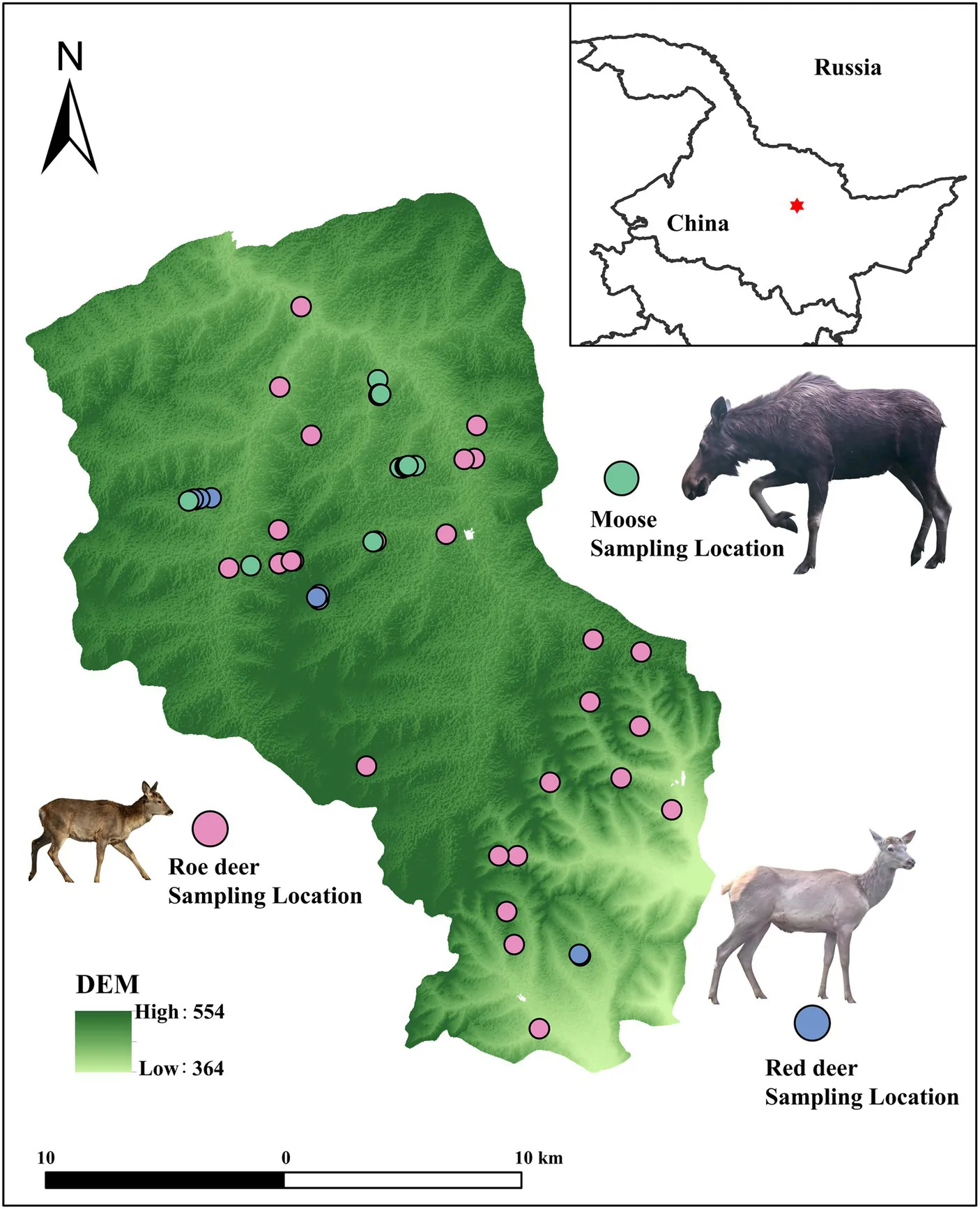
Study area and sampling locations. Green, blue, and pink circles indicate sampling locations for moose, red deer, and roe deer, respectively. The underlying DEM elevation dataset is provided by the Geospatial Data Cloud site, Computer Network Information Center, Chinese Academy of Sciences. (http://www.gscloud.cn).

### 2.2 DNA extractions

Total genomic DNA used for both bacterial microbiome and plant dietary analyses was extracted from each fecal sample using the E.Z.N.A.^®^ Stool DNA Kit (Omega Bio-tek, Norcross, GA, USA) following the manufacturer’s protocols. DNA integrity was assessed by 1% agarose gel electrophoresis, and DNA concentration and purity were evaluated using a NanoDrop 2000 spectrophotometer (Thermo Scientific, USA). All extracted DNA was stored at −80 °C until downstream PCR amplification.

### 2.3 PCR amplification and sequencing

The full-length bacterial 16S rRNA gene was amplified using universal primers 27F (5′-AGRGTTYGATYMTGGCTCAG-3′) and 1492R (5′-RGYTACCTTGTTACGACTT-3′) (Weisburg et al., 1991). PacBio barcode sequences were added to the 5′ end of each primer to distinguish individual samples. The 20 μL PCR system contained 4 μL of 5 × FastPfu buffer, 2 μL of 2.5 mM dNTPs, 0.8 μL forward primer (5 μM), 0.8 μL reverse primer (5 μM), 0.4 μL FastPfu DNA Polymerase, 10 ng template DNA, and DNase-free water to volume. PCR was performed on a T100 Thermal Cycler (BIO-RAD, USA) with the following program: initial denaturation at 95 °C for 3 min; 27 cycles of 95 °C for 30s, 60 °C for 30s, 72 °C for 45 s; a final extension at 72 °C for 10 min, followed by incubation at 4 °C. Amplified products were separated by agarose electrophoresis, purified with AMPure ^®^PB beads (Pacific Biosciences, CA, USA), and quantified using a Qubit 4.0 fluorometer (Thermo Fisher Scientific, USA). Equimolar purified amplicons were pooled, and SMRTbell libraries were constructed with the SMRTbell Prep Kit 3.0 (Pacific Biosciences, CA, USA) per official guidelines. Library sequencing was carried out on the PacBio Sequel IIe System at Majorbio Bio-Pharm Technology Co., Ltd. (Shanghai, China). Circular consensus sequencing was performed using SMRT Link v11.0 to generate high-fidelity (HiFi) reads from the raw subreads.

For plant dietary metabarcoding, a minibarcode region of the chloroplast rbcL gene was amplified using the primer pair rbcLminibarcodeF (5′-CTTACCAGYCTTGATCGTTACAAAGG-3′) and rbcLminibarcodeR (5′-GTAAAATCAAGTCCACCRCG-3′) on a T100 Thermal Cycler (Bio-Rad, USA) (Erickson et al., 2017). The 20 μL PCR mixture included 4 μL 5 × FastPfu buffer, 2 μL 2.5 mM dNTPs, 0.8 μL of each 5 μM primer, 0.4 μL FastPfu polymerase, 10 ng template DNA, and ddH₂O to volume. Thermal cycling conditions were set as: initial denaturation at 95 °C for 3 min; 27 cycles of 95 °C for 30 s, 55 °C for 30 s, 72 °C for 45 s; terminal extension at 72 °C for 10 min, then held at 4 °C. Target PCR fragments were recovered from 2% agarose gels and purified with the PCR Clean-Up Kit (YuHua, Shanghai, China). Purified products were quantified on a Qubit 4.0 fluorometer, mixed at equal molar concentrations, and subjected to paired-end sequencing (2 × 300 bp) on the Illumina NextSeq 2000 platform (Illumina, San Diego, CA, USA) by Majorbio Bio-Pharm Technology Co., Ltd. (Shanghai, China), following standard Illumina library preparation workflows. A no-template negative control was included in each PCR batch to monitor potential contamination.

### 2.4 Sequence processing and taxonomic assignment

PacBio HiFi reads were subjected to downstream sequence processing. Sequences were clustered into operational taxonomic units (OTUs) at 97% sequence similarity using UPARSE v11.0.667, with chimeric sequences removed during OTU clustering. Representative OTU sequences were taxonomically assigned against the NT_Taxon_core_v2024/16s_bacteria reference database using the classify-consensus-blast method based on BLAST.

For plant rbcL metabarcoding data, raw paired-end reads were quality-filtered using fastp v0.23.4 and merged using FLASH v1.2.11. Bases at the 3′ end were trimmed when the mean quality score within a 10-bp sliding window was below 20. Reads shorter than 50 bp after trimming or containing ambiguous bases were removed. Paired-end reads were merged with a minimum overlap of 10 bp and a maximum mismatch ratio of 0.2. Samples were demultiplexed according to barcode and primer sequences, allowing no barcode mismatches and a maximum of two primer mismatches. Quality-filtered sequences were clustered into OTUs at 97% sequence similarity using UPARSE v11.0, with chimeric sequences removed. Representative OTU sequences were taxonomically assigned against the nt_v20230830 database using BLAST.

### 2.5 Statistical data analysis

All statistical analyses and visualizations were performed in R v4.5.2 unless otherwise specified. Alpha diversity was evaluated separately for gut bacterial and plant dietary communities using rarefied abundance tables. Plant dietary samples were rarefied to 62,195 reads per sample, whereas gut bacterial samples were rarefied to 10,040 reads per sample. For bacterial communities, the Chao1 richness estimator and Shannon–Wiener diversity index were calculated. For plant dietary communities, observed genus richness, Chao1, Shannon, and Simpson diversity indices were calculated. Differences in alpha-diversity indices among moose, red deer, and roe deer were assessed using Kruskal–Wallis tests, followed by pairwise Wilcoxon rank-sum tests with Benjamini–Hochberg (BH) correction for multiple comparisons.

For beta-diversity analyses, genus-level abundance tables were converted to within-sample relative abundances to account for differences in sequencing depth. Bray–Curtis dissimilarities were then calculated, and principal coordinate analysis (PCoA) was used to visualize differences in community composition among the three host species. Overall differences among host species were evaluated using both analysis of similarities (ANOSIM) and permutational multivariate analysis of variance (PERMANOVA), with 999 permutations.

For community composition analyses, genus-level plant abundance matrices and phylum- and genus-level bacterial abundance matrices were converted to relative abundances. For visualization, the 20 most abundant plant genera, the 10 most abundant bacterial phyla, and the 20 most abundant bacterial genera were displayed, while the remaining low-abundance taxa were grouped as “Others.” Species-level mean relative abundances were visualized using stacked bar plots. Venn diagrams were used to visualize plant and bacterial taxa shared among or uniquely detected in the three host species.

The relative abundances of Bacillota and Bacteroidota were extracted from the phylum-level bacterial abundance table, and the Bacillota/Bacteroidota ratio was calculated for each sample. Differences in this ratio among the three host species were assessed using the Kruskal–Wallis test, followed by pairwise Wilcoxon rank-sum tests with BH correction for multiple comparisons.

Linear discriminant analysis effect size (LEfSe) was used to identify bacterial genera showing significant differences in relative abundance among host species (Segata et al., 2011). Differential taxa were identified using an LDA score threshold of > 3.0 and a significance threshold of *p* < 0.05.

PICRUSt2 was used to infer the functional potential of gut bacterial communities from 16S rRNA gene profiles (Douglas Gavin et al., 2020). Representative OTU sequences were phylogenetically placed using EPA-ng and GAPPA, and gene-family copy numbers were inferred using hidden-state prediction. Predicted gene-family abundances were corrected for 16S rRNA gene copy number and summarized into KEGG Ortholog (KO) profiles. KO abundances were subsequently summarized according to KEGG hierarchical functional categories, including Level 1 and Level 2 pathways, for downstream comparative analyses.

Predicted KEGG pathway abundances were converted to within-sample relative abundances (%). Differences in predicted pathway abundance among host species were evaluated using non-parametric tests. For Level 1 functional categories, pairwise comparisons were performed using Wilcoxon rank-sum tests with Benjamini–Hochberg (BH) correction for multiple comparisons. For Level 2 pathways, pairwise Wilcoxon rank-sum tests were performed for all pathways and all three host-species comparisons, and the resulting *p* values were corrected globally using the BH false-discovery-rate procedure. For visualization, Level 2 pathways were retained when the maximum mean relative abundance across the three host species was ≥1.0% and at least one pairwise comparison had an FDR-adjusted *p* < 0.05.

Concordance between plant dietary composition and gut bacterial community structure was evaluated using Procrustes analysis. Bray–Curtis dissimilarity matrices were calculated separately for genus-level dietary and bacterial communities and subjected to principal coordinate analysis. The resulting PCoA configurations were compared using Procrustes rotation, and statistical significance was evaluated using the PROTEST permutation test implemented in the vegan package with 999 permutations. Procrustes analyses were conducted both across all samples and separately within each host species. The Procrustes residual sum of squares (M^2^) and permutation-based *p* values were used to characterize the degree and significance of concordance between dietary and bacterial community configurations.

Redundancy analysis (RDA) was used to explore associations between dietary plant composition and gut bacterial community structure. Prior to RDA, bacterial genus-level abundance data were Hellinger-transformed. To reduce model dimensionality and the risk of overfitting, dietary plant genera were ranked according to their total abundance, and the 10 most abundant genera were retained as candidate explanatory variables. Multicollinearity among explanatory variables was evaluated using variance inflation factors (VIFs), with variables showing VIF > 10 removed iteratively before final model fitting. The significance of the overall RDA model was evaluated using permutation ANOVA with 999 permutations, and adjusted R^2^ was calculated to estimate the proportion of bacterial community variation explained by dietary variables. To evaluate whether the unequal sample size, particularly the small red deer group (*n* = 7), affected the overall result, a sensitivity analysis was additionally conducted by repeating the global RDA after excluding all red deer samples (*n* = 48).

## 3 Result

### 3.1 Sequencing results

A total of 55 fresh fecal samples collected from three cervid species were subjected to plant dietary metabarcoding and 16S rRNA gene sequencing analyses. After strict quality control and sequence optimization, 2,645 OTUs were identified from 2,958,954 raw reads for the 16S rRNA sequencing dataset, with an average sequencing depth of 53799.16 ± 18261.45 reads per sample. For plant dietary metabarcoding, 1,663 OTUs were obtained from 6,643,689 raw reads, and the average sequencing depth per sample was 105455.38 ± 24371.85 reads.

### 3.2 Dietary composition and abundance analysis

Venn diagram analysis at the genus level was performed to identify shared and unique food genera among moose, red deer and roe deer (Figure 2a). A total of 16 plant genera were consumed by all three cervid species, accounting for 12.60% of all detected food genera. Moose possessed 28 unique food genera (22.05%), whereas roe deer harbored the greatest number of unique food genera (47, 37.01%). No unique food genera were detected in red deer. The genera shared only by moose and red deer, moose and roe deer, red deer and roe deer were 15 (11.81%), 4 (3.15%) and 17 (13.39%), respectively. We further calculated the genus-level relative dietary abundance based on sequencing results, and the top 20 genera were selected to generate the stacked bar chart (Figure 2b). *Betula* was the dominant food genus shared by all three species (moose: 65% ± 22%, red deer: 27% ± 32%, roe deer: 39% ± 29%). Besides *Betula*, moose also heavily relied on *Alnus* (25% ± 17%). The dominant food resources of red deer consisted of *Tilia* (24% ± 30%), *Euonymus* (14% ± 22%) and *Filipendula* (12% ± 17%). Roe deer exhibited a diverse assemblage of dominant food genera, including *Carex* (9% ± 19%), *Filipendula* (9% ± 11%), *Rhododendron* (7% ± 19%), *Cornus* (5% ± 18%) and *Euonymus* (5% ± 12%).

**Figure 2:**
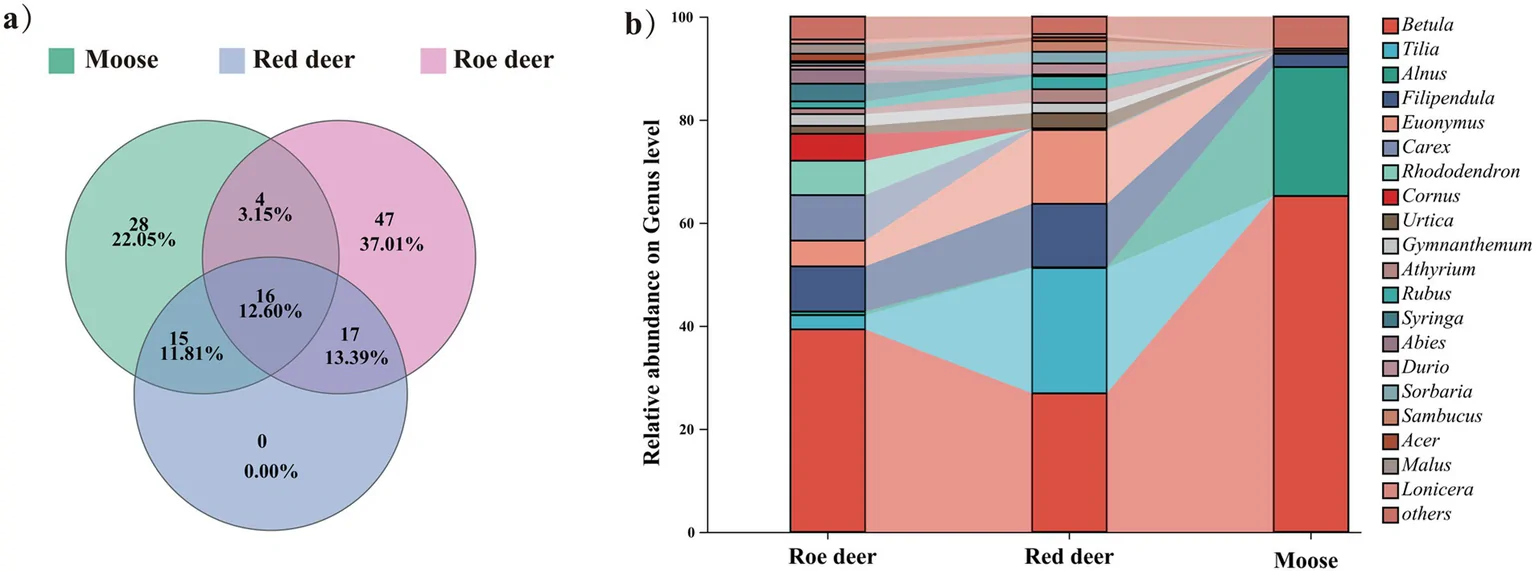
Overview of the dietary habits of three sympatric cervid species. **(a)** Venn diagram showing shared and unique food resources among the three cervid species. **(b)** Bar plot displaying the relative abundance of dietary plant genera at the genus level for three cervid species.

### 3.3 Analysis of dietary alpha and beta diversity

Dietary alpha diversity differed significantly among the three cervid species. Shannon diversity differed significantly among species (Kruskal–Wallis: χ^2^ = 17.929, *p* < 0.001), with both red deer (BH-adjusted *p* = 0.002) and roe deer (BH-adjusted *p* < 0.001) showing higher Shannon diversity than moose, whereas red deer and roe deer did not differ significantly (BH-adjusted *p* = 0.660). Chao1 richness also differed significantly among species (Kruskal–Wallis: χ^2^ = 10.493, *p* = 0.005), but pairwise comparisons showed a significant difference only between moose and roe deer (BH-adjusted *p* = 0.006); the moose–red deer and red deer–roe deer comparisons were not significant (Figures 3a,b). Beta-diversity analysis based on Bray–Curtis dissimilarities showed substantial overlap between red deer and roe deer, whereas moose were more clearly separated from the other two species in the PCoA ordination (Figure 3c). This interspecific differentiation in dietary plant composition was supported by both ANOSIM (*R* = 0.227, *p* = 0.001) and PERMANOVA (pseudo-*F* = 7.429, *R^2^* = 0.222, *p* = 0.001).

**Figure 3:**
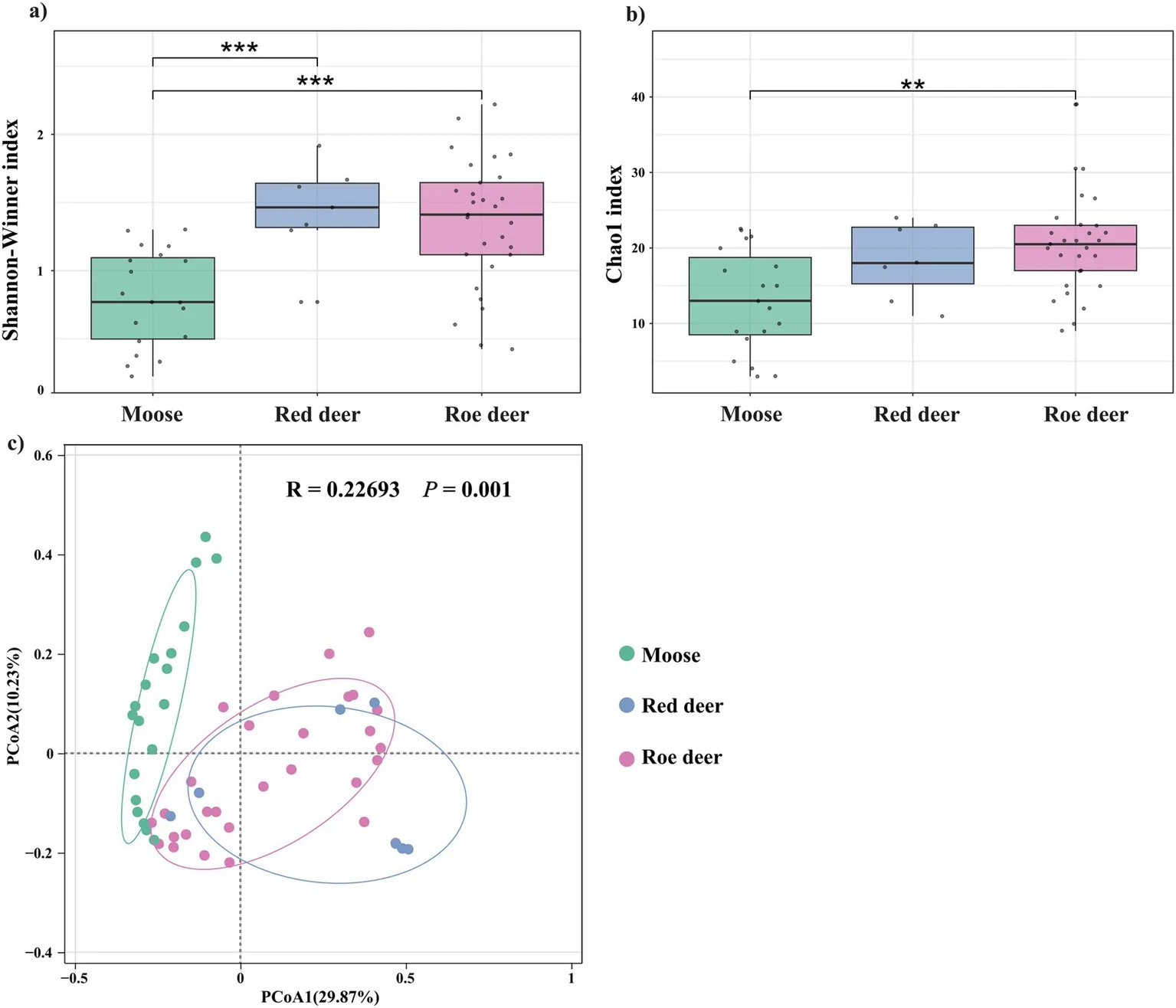
Dietary alpha and beta diversity of three cervids at genus level. **(a)** Box plot displaying the Shannon index. **(b)** Box plot displaying the Chao1 index. **(c)** PCoA ordination plot for beta diversity analysis. * adjusted *p* < 0.05, ** adjusted *p* < 0.01, *** adjusted *p* < 0.001.

### 3.4 Microbiome composition and abundance analysis

Based on high-throughput sequencing data, we quantified the relative abundance of gut microbiota in three cervid species at both the phylum and genus levels. The top 10 dominant bacterial phyla and top 20 bacterial genera were selected and visualized using stacked bar charts (Figures 4c,d). At the phylum level, Bacillota (moose: 81% ± 9%, red deer: 80% ± 7%, roe deer: 81% ± 5%) and Bacteroidota (moose: 6% ± 3%, red deer: 13% ± 6%, roe deer: 14% ± 5%) dominated the gut microbial communities of all three cervids. At the genus level, a total of 25 bacterial genera exhibited a relative abundance greater than 1% across all three species. We further compared the interspecific differences in the Bacillota/Bacteroidota abundance ratio among the three cervids (Figure 4b). The results showed that moose exhibited a significantly higher Bacillota/Bacteroidota ratio than red deer and roe deer, whereas no significant difference was observed between red deer and roe deer.

**Figure 4:**
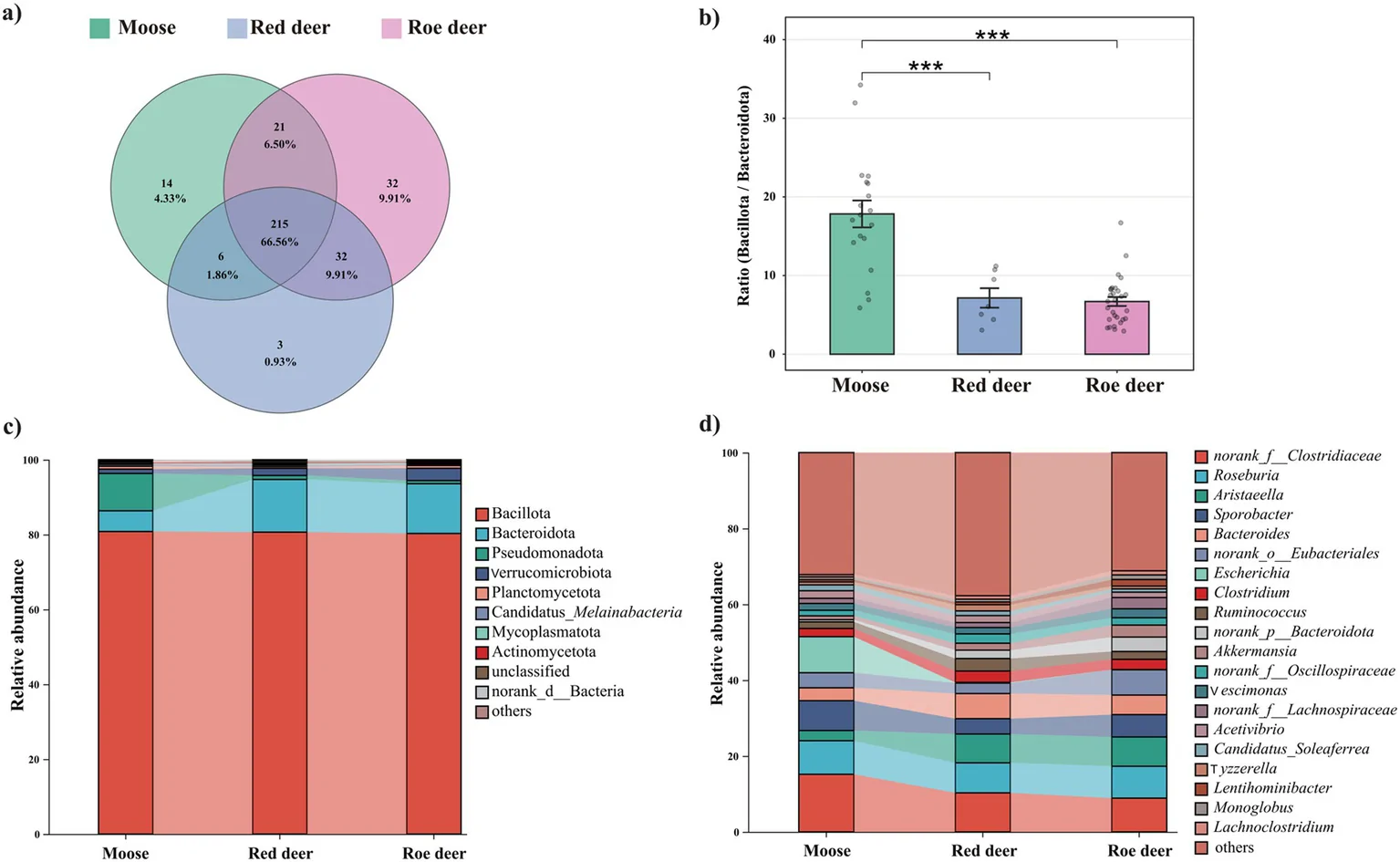
Overview of gut microbiome composition in three sympatric cervid species. **(a)** Venn diagram showing shared and unique genera at the genus level. **(b)** Bar plot showing the Bacillota/Bacteroidota ratio among the three cervid species, with mean ± SEM and individual sample values. **(c)** Stacked bar charts displaying the relative abundance of the top 10 phyla. **(d)** Stacked bar charts displaying the relative abundance of the top 20 genera. * adjusted *p* < 0.05, ** adjusted *p* < 0.01, *** adjusted *p* < 0.001.

Venn diagram analysis was performed to characterize the shared and unique bacterial genera among the three cervid species at the genus level (Figure 4a). In total, 215 bacterial genera were shared by moose, red deer, and roe deer, accounting for 66.56% of all detected genera. In terms of species-specific bacterial genera, moose harbored 14 unique genera (4.33%), roe deer harbored the highest number of unique genera (32 genera, 9.91%), and red deer had only three unique genera (0.93%). For pairwise shared genera, 21 genera (6.50%) were exclusively shared between moose and roe deer, six genera (1.86%) were uniquely shared between moose and red deer, and 32 genera (9.91%) were common only to red deer and roe deer.

### 3.5 Analysis of microbiome alpha and beta diversity

Gut bacterial alpha diversity also differed among the three cervid species. The Shannon–Wiener index differed significantly among species (Kruskal–Wallis: χ^2^ = 27.127, *p* < 0.001). Pairwise comparisons showed that roe deer had higher Shannon diversity than both moose (BH-adjusted *p* < 0.001) and red deer (BH-adjusted *p* = 0.042), while red deer also had higher Shannon diversity than moose (BH-adjusted *p* = 0.042). In contrast, no significant interspecific difference was detected in Chao1 richness (Kruskal–Wallis: χ^2^ = 3.485, *p* = 0.175; Figures 5a,b). Beta-diversity analysis based on Bray–Curtis dissimilarities showed clear differentiation in gut bacterial community structure among the three cervid species, with moose forming the most distinct cluster and red deer and roe deer showing greater overlap in the PCoA ordination (Figure 5c). This interspecific differentiation was supported by both ANOSIM (*R* = 0.789, *p* = 0.001) and PERMANOVA (pseudo-*F* = 12.619, *R^2^* = 0.327, *p* = 0.001). To further identify discriminatory gut microbial biomarkers associated with community differences among the three groups, we performed LEfSe analysis at the genus level and screened 16 bacterial biomarkers with an LDA threshold of 3. Of all differentially abundant genera identified by LEfSe, *Escherichia* yielded the maximum LDA score and represented the primary taxonomic biomarker that discriminated moose from the other two cervid species. The taxon unclassified_f__Lachnospiraceae was the dominant signature genus for roe deer, while norank_p__Bacteroidota displayed the highest discriminatory capacity to separate the gut microbiota of red deer (Figure 5d).

**Figure 5:**
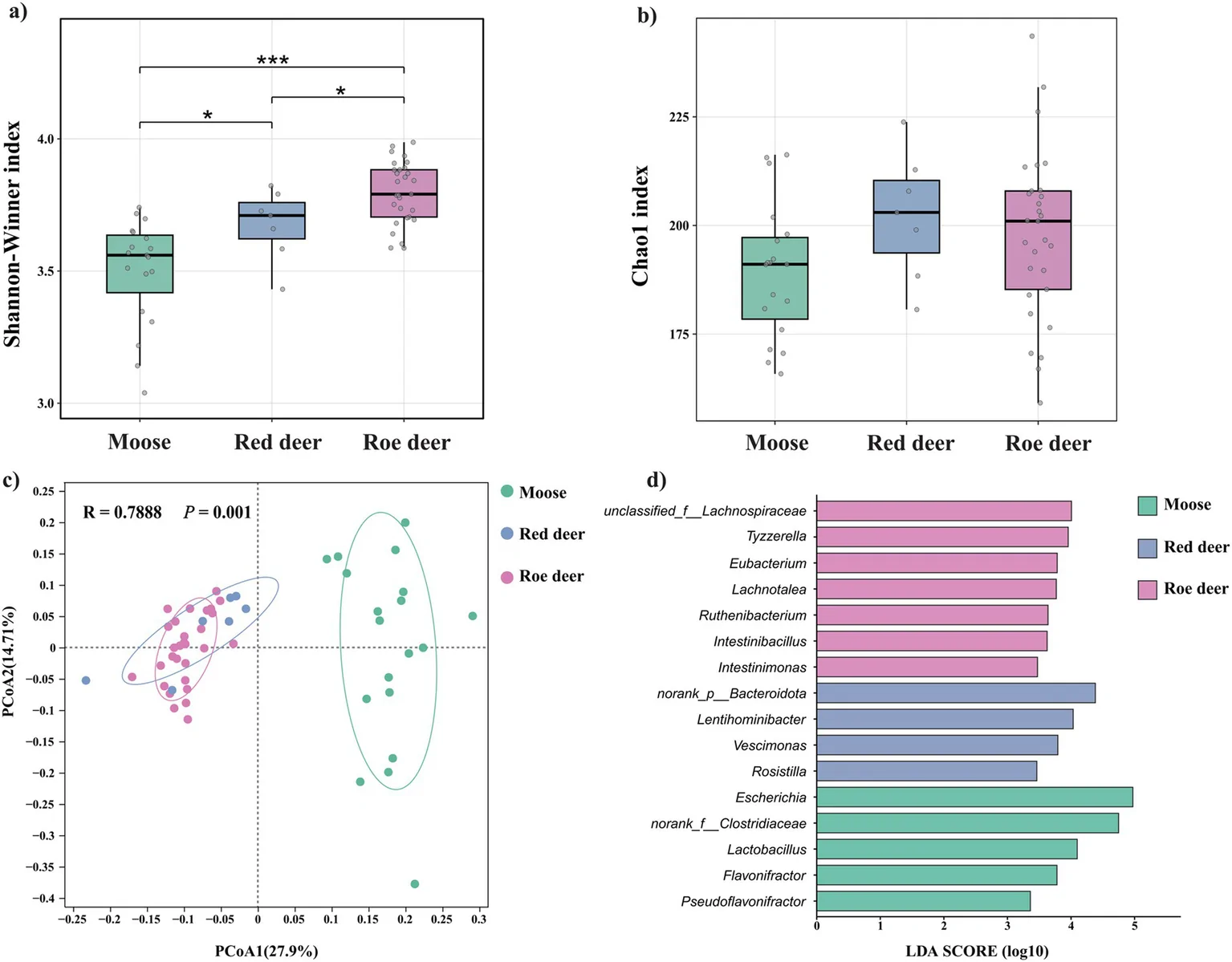
Overview of genus-level alpha and beta diversity and microbial biomarkers of gut microbiome in three sympatric cervid species. **(a)** Box plot showing the Shannon-Wiener index. **(b)** Box plot displaying the Chao1 index. **(c)** PCoA ordination plot for beta diversity analysis. **(d)** LDA bar plot generated by LEfSe analysis. * adjusted *p* < 0.05, ** adjusted *p* < 0.01, *** adjusted *p* < 0.001.

### 3.6 Predicted functional analysis of the microbiome

To investigate differences in the predicted functional potential of gut microbiomes among the three cervid species, we inferred functional profiles using the Kyoto Encyclopedia of Genes and Genomes (KEGG) database and visualized the relative abundance of level-1 functional categories with box plots (Figure 6). Moose showed significantly higher relative abundances of Cellular Processes, Environmental Information Processing, and Human Diseases than both red deer and roe deer, whereas Metabolism and Organismal Systems were significantly lower in moose than in the other two species. Genetic Information Processing was also lower in moose than in red deer and roe deer, and a smaller but significant difference was detected between red deer and roe deer.

**Figure 6:**
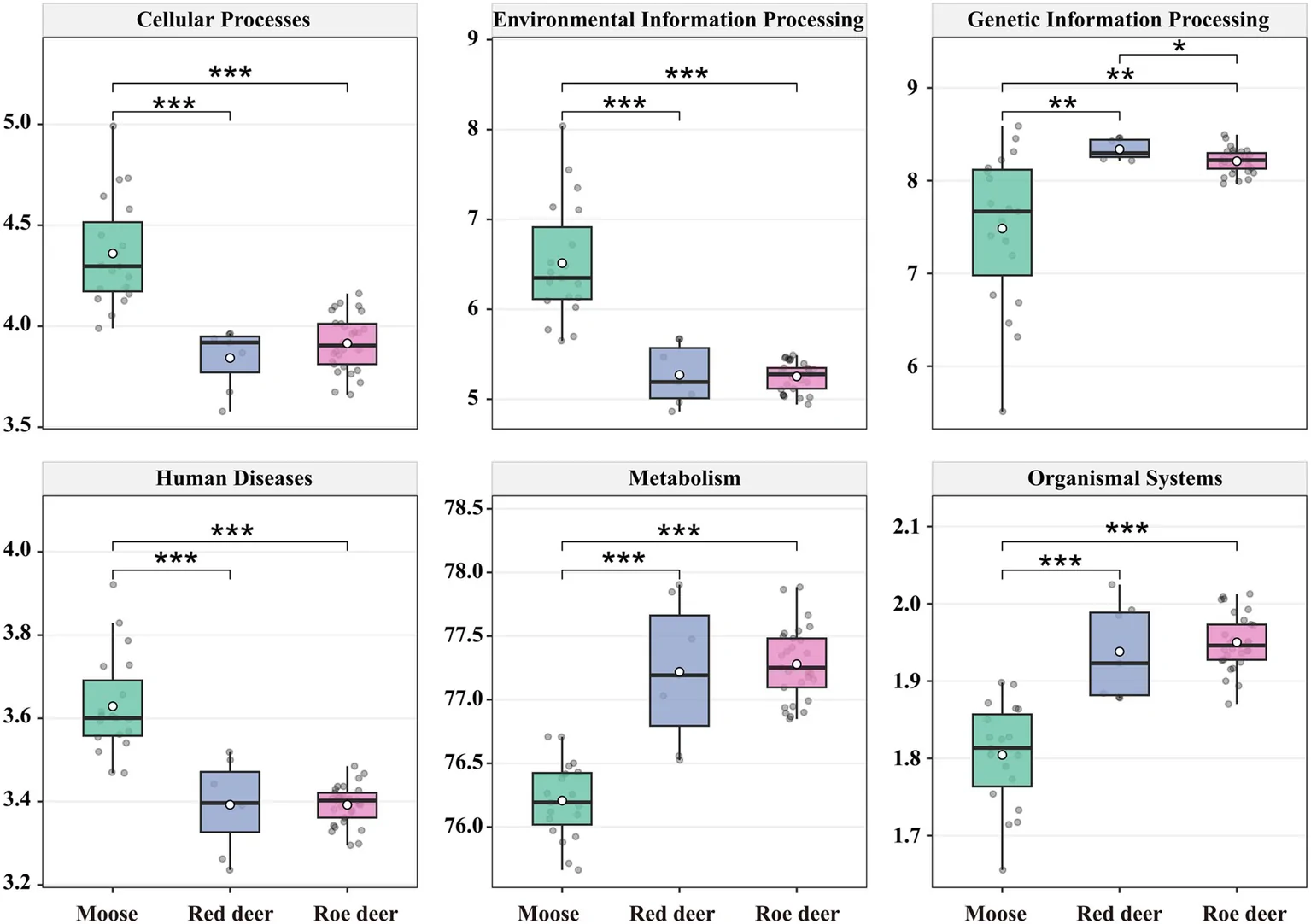
Overview of predicted level-1 KEGG functional pathway profiles of gut microbiota in three sympatric cervid species. * adjusted *p* < 0.05, ** adjusted *p* < 0.01, *** adjusted *p* < 0.001.

To further characterize differences in predicted microbial functional potential among the three sympatric cervid species, we compared the relative abundance of level-2 KEGG sub-pathways across moose, red deer, and roe deer using box plots (Figure 7). At KEGG Level 2, 15 pathways met the predefined criteria of a maximum mean relative abundance ≥1.0% and at least one significant pairwise comparison after global BH-FDR correction. Moose showed significantly higher predicted abundances of Cell motility, Cellular community–prokaryotes, Drug resistance: antimicrobial, Membrane transport, Metabolism of other amino acids, and Signal transduction than both red deer and roe deer. In contrast, Amino acid metabolism, Biosynthesis of other secondary metabolites, Folding, sorting and degradation, Global and overview maps, Glycan biosynthesis and metabolism, and Translation were significantly lower in moose than in both red deer and roe deer. Replication and repair was also lower in moose than in both species, although the differences were weaker. Energy metabolism differed significantly only between moose and roe deer, with a higher relative abundance in moose, whereas Lipid metabolism was significantly higher in roe deer than in moose. Translation was the only displayed Level 2 pathway showing significant differences among all three pairwise comparisons.

**Figure 7:**
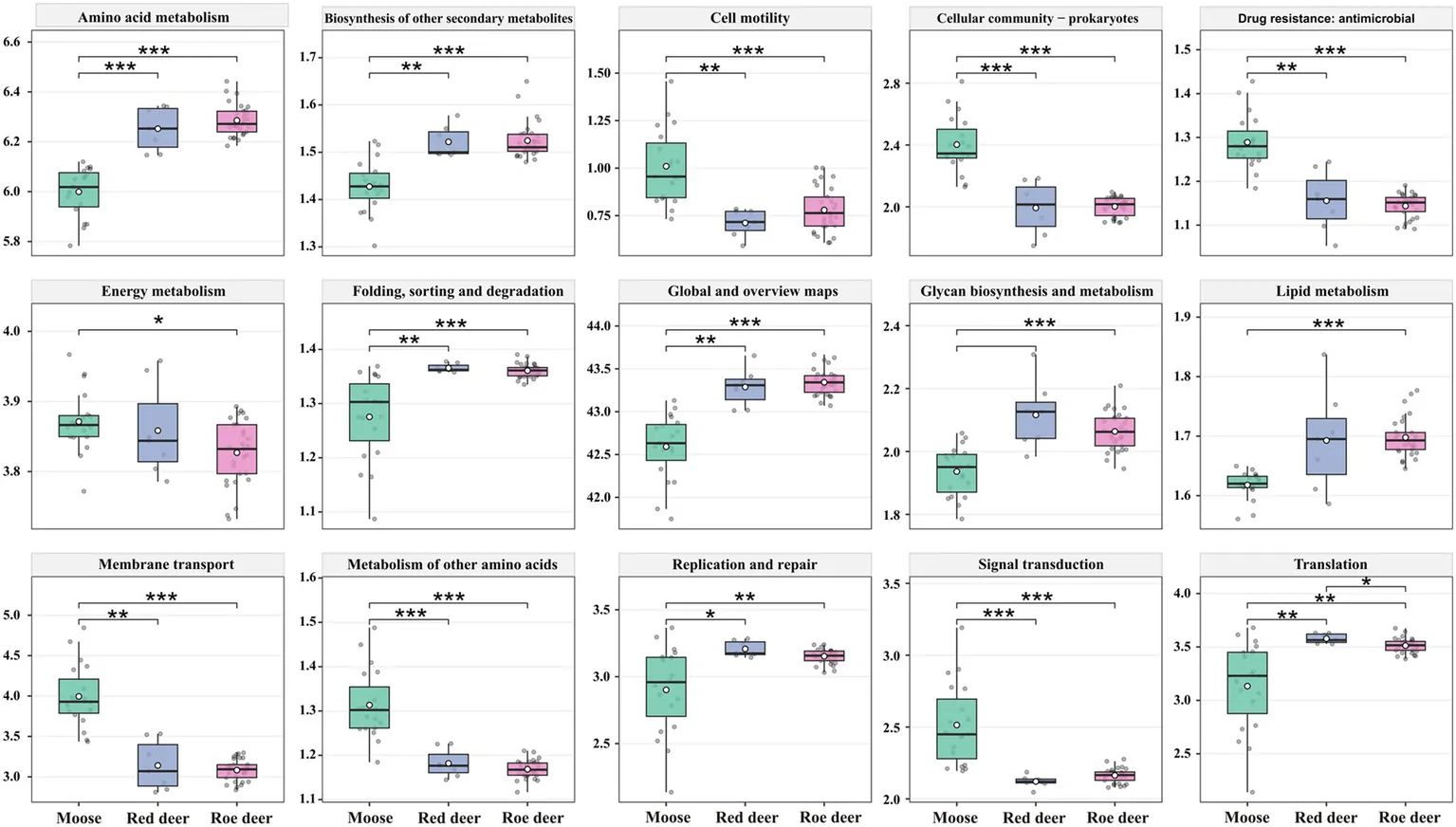
Overview of predicted level-2 KEGG functional pathway profiles of gut microbiota in three sympatric cervid species. * adjusted *p* < 0.05, ** adjusted *p* < 0.01, *** adjusted *p* < 0.001.

### 3.7 Associations between dietary composition and gut microbiome structure

To reveal the correlation between diet composition and gut microbial communities of three sympatric cervid species, we performed Procrustes analysis and redundancy analysis (RDA). Procrustes analysis revealed a significant association between dietary plant composition and gut microbiota structure (M^2^ = 0.629, *p* = 0.001, Figure 8a), indicating coordinated variation between dietary and bacterial community composition across samples. RDA further identified dietary plant taxa shaping the gut bacterial assembly of each cervid species (Adj. R^2^ = 0.223, *p* = 0.001, Figure 8b). *Alnus* and *Betula* were strongly associated with the gut microbiota of moose. *Tilia* was the primary dietary factor driving the microbial differentiation of red deer, while multiple taxa including *Filipendula*, *Carex*, *Euonymus*, *Cornus*, *Abies*, *Syringa* and *Rhododendron* closely correlated with the gut community of roe deer. All retained variables had VIF values below 10, indicating no excessive multicollinearity among the dietary predictors.

**Figure 8:**
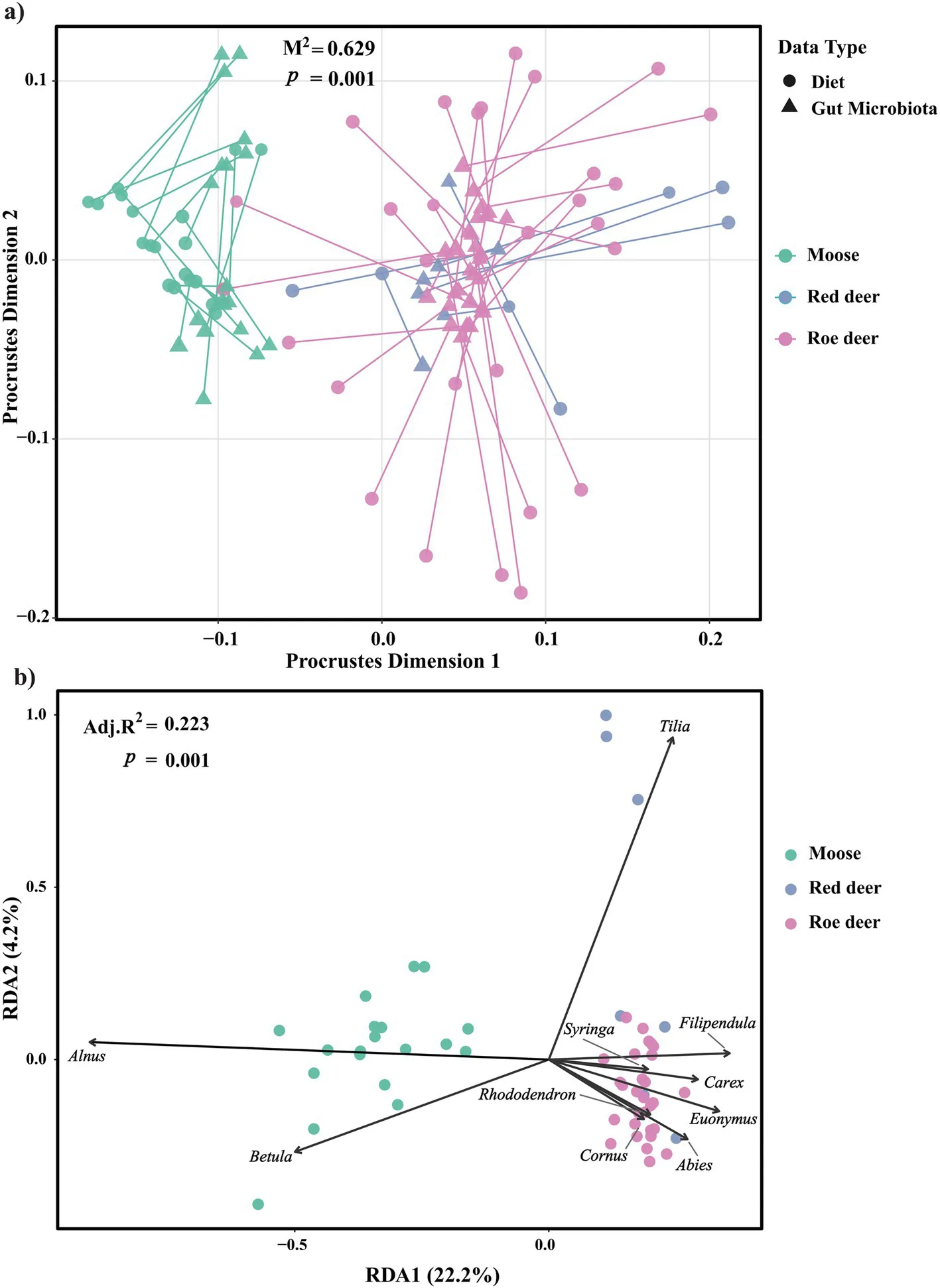
Overview of the correlation between dietary and gut microbiota in three sympatric cervid species. **(a)** Procrustes analysis plot of diet and gut microbial communities. **(b)** RDA ordination plot showing the associations between dominant dietary plant genera and gut microbial communities.

To assess the potential influence of the smaller red deer sample, we repeated the global RDA after excluding all seven red deer samples. The association remained significant (*n* = 48, Adj. R*^2^* = 0.231, *F* = 2.409, *p* = 0.001), with a similar proportion of explained variation to that obtained from the complete dataset.

## 4 Discussion

This study used plant DNA metabarcoding and 16S rRNA high-throughput sequencing of the gut microbiota to investigate differences between species in the composition of forage resources, the structure and predicted function of the gut microbial community of three sympatric cervid species: moose, red deer, and roe deer. Furthermore, the research examined how these disparities, alongside the interactions between diet and gut microbiota, contribute to niche differentiation.

### 4.1 Dietary differentiation among sympatric moose, red deer and roe deer

Consistent with classical niche differentiation theory (Schoener, 1974), our DNA metabarcoding data reveal clear trophic resource partitioning among the three sympatric cervid species. All three taxa share a core dietary pool comprising 16 plant genera, with *Betula* acting as the dominant shared food source, which indicates partial overlap of their trophic niches within the shared forest habitat. Nevertheless, the three cervids exhibit pronounced divergence in both the taxonomic composition of their diets and the functional plant groups they primarily exploit.

Roe deer harboured the largest set of unique food genera, composed primarily of low shrubs, forbs and graminoids such as *Carex*, *Rhododendron* and *Cornus*, reflecting exploitation of understorey resources rarely used by the larger cervids. Moose primarily feed on woody shrubs exemplified by Alnus, which together with Betula accounted for approximately 90% of its total dietary intake. In marked contrast, red deer had no unique food genera, indicating that it achieves dietary separation entirely by adjusting intake proportions of shared resources rather than relying on exclusive forage taxa. Pairwise sharing patterns further mirrored this functional divergence: moose and red deer shared 15 woody genera, whereas red deer and roe deer shared 17 genera dominated by shrubs and herbs, resulting in higher dietary similarity between the latter pair.

These compositional differences were further confirmed by an evaluation of their dietary alpha diversity. Shannon–Wiener diversity was significantly lower in moose than in both red deer and roe deer, whereas no significant difference was detected between red deer and roe deer. Chao1 richness showed a different pattern: roe deer had significantly higher richness than moose, whereas red deer did not differ significantly from either moose or roe deer. This pattern is consistent with the intermediate dietary strategy of red deer, whose dietary richness overlapped with both the more specialized moose and the more diverse roe deer. As in previous studies, moose primarily rely on woody plants, and their highly specialised diet concentrates their foraging on a few species of high-fibre shrubs (Kuijper et al., 2016; Spitzer et al., 2024). This results in low dietary richness and evenness. In contrast, roe deer utilized a broader range of woody plants, shrubs, and herbaceous plants, resulting in greater dietary richness and diversity, while red deer occupied an intermediate position. Beta-diversity analysis further corroborated species-level dietary differentiation at the whole-community scale. PCoA ordination showed a distinct cluster for moose separated from the other two species, whereas red deer and roe deer exhibited substantial overlap in dietary community space.

Collectively, these results demonstrate that the three sympatric cervids have evolved distinct dietary niches: moose function as narrow-niche woody specialists, roe deer as broad-niche mixed feeders exploiting diverse understorey vegetation, and red deer as an intermediate strategist that partitions resources primarily via proportional shifts rather than specific species. This is primarily shaped by body size and associated digestive constraints, consistent with the Jarman-Bell principle (Jarman, 1974; Demment and Soest, 1985). The Jarman-Bell principle states that there is a correlation between body size and food quality: larger species consume more low-quality food, while smaller herbivores tend to select higher-quality food resources (Jarman, 1974).

### 4.2 Associations between dietary differentiation and microbial community structure and predicted function across three cervids

Dietary composition is widely recognized as an important factor associated with gut microbial composition and diversity (Rinninella et al., 2019). In the present study, Bacillota and Bacteroidota dominated the gut microbiota of all three cervid species, consistent with patterns widely reported in wild ruminants and other herbivorous mammals (Gao et al., 2020; Jiang et al., 2022; Zhang et al., 2024). Nevertheless, the relative representation of these major phyla differed among hosts. Moose exhibited the highest Bacillota/Bacteroidota ratio, whereas roe deer showed the lowest ratio and red deer occupied an intermediate position. A higher Bacillota/Bacteroidota ratio has been associated with microbial communities involved in the utilization of structural carbohydrates and energy extraction from fibrous diets (Ma et al., 2019). This pattern corresponded with the highly specialized woody diet of moose, which was dominated by lignified shrubs and trees such as Betula and Alnus. In contrast, the relatively greater representation of Bacteroidota in roe deer may be consistent with its broader use of shrubs, forbs, and graminoids, because members of this phylum include taxa with capacities for utilizing pectin and other soluble polysaccharides (Martens et al., 2011; Flint et al., 2012; Seveso et al., 2024). Red deer again showed an intermediate microbial composition, paralleling its mixed feeding strategy based largely on proportional use of shared food resources. The comparatively large body size of moose is also associated with greater absolute energetic requirements under general metabolic scaling relationships (Silva et al., 2006), although the present observational data do not allow these physiological characteristics to be directly linked to the observed microbial differences.

Microbial diversity showed a similar, although not identical, correspondence with dietary patterns. Roe deer exhibited the highest Shannon–Wiener diversity, whereas moose showed the lowest Shannon diversity and red deer occupied an intermediate position. In contrast, Chao1 richness did not differ significantly among the three cervid species. Previous studies have likewise reported associations between dietary diversity and gut microbial diversity, potentially because heterogeneous dietary substrates can provide a wider range of ecological niches for microbial taxa (Reese and Dunn, 2018; Li et al., 2021; Dahl et al., 2023). At the community level, gut bacterial composition also differed among the three cervids, with moose showing the most distinct community configuration. Together, these patterns indicate that the cervid species exhibiting the strongest dietary specialization also showed the clearest microbial differentiation, supporting a correspondence between trophic niche differentiation and gut microbial community structure.

LEfSe analysis and PICRUSt2-based KEGG prediction further indicated that taxonomic differentiation among the three gut microbial communities was accompanied by differences in predicted metabolic potential. Moose showed enrichment of several bacterial biomarkers, including *Escherichia* and norank_f__Clostridiaceae. At the predicted functional level, several pathways related to cellular processes and signal transduction were more abundant in moose, whereas Replication and repair was lower in moose than in red deer and roe deer. Energy metabolism differed only modestly among species and was significantly higher in moose than in roe deer. Previous work has shown that microbial taxa inhabiting herbivore digestive systems can display adaptations related to carbohydrate utilization and the rumen environment (Flint et al., 2012; Pang et al., 2020; Dahl et al., 2023). In roe deer, bacterial biomarkers associated in previous studies with carbohydrate utilization and processing of diverse dietary substrates were comparatively enriched, while predicted pathways involving glycan biosynthesis and metabolism, metabolism of other amino acids, and biosynthesis of other secondary metabolites were also more abundant. These predicted functional differences are broadly consistent with the more diverse plant substrates and secondary compounds present in the roe deer diet (Cotta, 1992; Gardner et al., 1995; Flint et al., 2012; Dahl et al., 2023). Red deer exhibited a comparatively intermediate taxonomic and predicted functional profile, corresponding to its intermediate dietary strategy based primarily on proportional differences in shared food resources rather than exclusive plant genera. Because these functional profiles were inferred from 16S rRNA gene data rather than measured directly, they should be interpreted as predicted metabolic potential rather than demonstrated microbial or host physiological function. Nevertheless, the parallel differentiation of dietary composition, bacterial taxa, and predicted functional profiles suggests that contrasting trophic strategies are accompanied by distinct microbial ecological configurations. A similar pattern has been reported in wild ruminants inhabiting severe foraging environments. Using 16S rRNA sequencing, metagenomics, and measurements of forage digestibility, researchers found that Tibetan antelope and blue sheep exhibited gut microbial characteristics associated with enhanced forage utilization under nutritionally constrained conditions, including enrichment of cellulolytic taxa and genes related to cellulose and hemicellulose degradation (Liu et al., 2025). These findings further support the ecological relevance of considering gut microbial variation together with dietary resources and foraging environments when evaluating trophic adaptation in wild ruminants.

Procrustes and RDA analyses further supported the correspondence between dietary composition and gut bacterial community structure in the present study. The significant Procrustes concordance indicated coordinated variation between dietary and bacterial community configurations, while the global RDA showed that variation in dietary plant composition was significantly associated with bacterial community variation across the complete dataset. Importantly, this relationship remained significant after exclusion of all red deer samples, indicating that the overall diet–microbiome association was not dependent on the smaller red deer group. These analyses therefore strengthen the evidence that dietary differentiation and microbial differentiation covary across the three sympatric cervids. However, because the study was observational, these associations should not be interpreted as evidence that dietary differences directly caused the observed microbial differences or that microbial differentiation itself caused trophic niche partitioning.

### 4.3 Summary

This study demonstrates that three sympatric cervids partition winter dietary resources and exhibit corresponding differentiation in fecal gut microbial communities. Moose showed the strongest trophic specialization, characterized by a woody-plant-dominated diet, lower dietary diversity, and the most distinct bacterial community structure. Roe deer displayed broader dietary resource use and higher dietary and microbial diversity, whereas red deer generally occupied an intermediate position. These parallel interspecific patterns indicate that variation in trophic strategy is closely associated with variation in gut microbial community composition and predicted metabolic potential.

The significant Procrustes concordance and global RDA provide direct statistical evidence that dietary plant composition and bacterial community structure covary across hosts. Importantly, this relationship remained significant after exclusion of the smaller red deer group, supporting the robustness of the overall diet–microbiome association despite unequal sample sizes. In combination with the taxonomic and predicted functional differences identified by LEfSe and PICRUSt2, these findings suggest that gut microbial differentiation represents an additional ecological dimension accompanying trophic niche partitioning among sympatric cervids.

From an ecological perspective, the coordinated differentiation of diet and gut microbiota may help explain how closely related herbivores occupying the same landscape maintain distinct trophic strategies. Rather than demonstrating a direct causal pathway, our results identify a consistent association between macroscopic resource partitioning and microscopic microbial differentiation. This association provides a useful framework for considering gut microbiota alongside host diet, body size, physiology, phylogeny, and habitat use when investigating mechanisms of sympatric coexistence. In this framework, microbial differentiation may reflect adaptation to contrasting dietary environments and could potentially contribute to differences in digestive capacity, but these possibilities require direct testing.

The present study was based on samples collected during a single winter period, and the red deer sample size was relatively small. Moreover, microbial functional profiles were predicted from 16S rRNA gene data rather than directly measured. Future studies using balanced longitudinal sampling, controlled dietary experiments, shotgun metagenomics, metabolomics, and measurements of digestive performance will be necessary to determine the direction and mechanisms of diet–microbiome interactions.

Nevertheless, by jointly characterizing dietary composition and gut microbiota from the same wild samples, this study demonstrates that interspecific differences in trophic strategy are paralleled by differences in gut microbial community structure and predicted functional potential. The significant concordance revealed by Procrustes analysis and global RDA further indicates that dietary and microbial differentiation are closely linked across the three sympatric cervids, providing a broader ecological perspective on trophic niche partitioning that incorporates both food-resource use and host-associated microbial variation.

## Data Availability

The raw sequencing data generated in this study have been deposited in the NCBI Sequence Read Archive under SRA Study accession SRP737642 and BioProject accession PRJNA1530993. Other datasets generated and analyzed during the current study are available from the corresponding author upon reasonable request.
